# The rising tide of drug-induced psychosis

**DOI:** 10.1007/s40211-025-00541-7

**Published:** 2025-08-20

**Authors:** Robin M. Murray, Alessandra Paparelli, Marta Di Forti, Paul Morrison

**Affiliations:** 1 Department of Psychosis Studies, Institute of Psychiatry, King’s College London, de Crespigny Park, SE5 8AF London, UK; 2 NHS Highland Health and Social Care Partnership, Lochgilphead, Scotland UK

**Keywords:** Recreational drug use, Schizophrenia, Epidemic, Methamphetamine, Cannabis, Drogenkonsum in der Freizeit, Schizophrenie, Epidemie, Methamphetamin, Cannabis

## Abstract

Recreational use of psychotogenic drugs has become more common in the last 30 years with consequent increase in drug-induced psychosis; 25–50% of such psychoses progress to schizophrenia within 5 years. Most cases result from abuse of methamphetamine or cannabis. The spread of methamphetamine psychosis has occurred without any liberalisation of laws; it is highly addictive and easy to make in small “kitchen” labs. The increase in cannabis use has been facilitated by a softening of attitudes towards the drug, and in some countries, especially in North America, by legalisation for so-called “medicinal use” and then recreational use. The potency of cannabis has greatly increased, especially in countries that have legalised its use, and the worldwide legal sales of cannabis for 2025 are estimated at $ 65 billion. The consequences are being seen with many reports of increases in the incidence of cannabis-psychosis and schizophrenia. In the 1980s some physicians predicted an epidemic of obesity and diabetes due to changes in diet and life style. Sadly, their predictions were correct. We are now at the beginning of a similar epidemic of drug-induced psychosis. Public education campaigns of the type that decreased tobacco smoking are necessary to avoid psychiatric services being overwhelmed.

## Abstract (de)

Der Freizeitkonsum psychomimetischer Drogen ist in den vergangenen 30 Jahren gestiegen und damit auch die Zahl der drogeninduzierten Psychosen. 25-50% dieser Psychosen entwickeln sich innerhalb von 5 Jahren zu einer Schizophrenie. Die meisten Fälle sind auf den Missbrauch von Methamphetamin oder Cannabis zurückzuführen. Zur Ausbreitung von Methamphetamin-Psychosen kam es ohne gesetzliche Liberalisierung. Methamphetamin hat ein ausgeprägtes Suchtpotenzial und kann unkompliziert in kleinen „Küchenlabors“ hergestellt werden. Der Anstieg des Cannabiskonsums wurde begünstigt durch weniger strikte und weniger autoritär gewordene Überzeugungen und Einstellungen, in einigen Ländern, insbesondere in Nordamerika, auch durch die Legalisierung des sog. „medizinischen Gebrauchs“ und anschließend die des rekreativen Konsums. Die Wirkungsstärke von Cannabis ist vor allem in den Ländern, die den Konsum legalisiert haben, deutlich gestiegen, und der weltweite legale Umsatz mit Cannabis wird für das Jahr 2025 auf 65 Mrd. Dollar geschätzt. Die Folgen zeigen sich in vielen Mitteilungen über einen Anstieg der Inzidenz cannabisinduzierten Psychosen und Schizophrenie. In den 1980er-Jahren sagten einige Ärzte eine Epidemie von Adipositas und Diabetes aufgrund von Veränderungen in Ernährung und Lebensstil vorher. Diese Vorhersagen haben sich leider bestätigt. Mit dem Anstieg der Inzidenz drogeninduzierter Psychosen stehen wir jetzt am Anfang einer ähnlichen Epidemie. Öffentliche Aufklärungskampagnen, wie die, mit denen der Tabakkonsum eingedämmt wurde, sind notwendig, um zu verhindern, dass die Psychiatrien und die psychiatrischen Dienste überlastet werden.

Swiss chemist Albert Hoffman, working for the pharmaceutical company Sandoz, synthesized lysergic acid diethylamide (LSD) in 1938 but he did not appreciate its psychotogenic properties until five years later when he accidentally spilt some on his skin. He recounted that he saw streams of “fantastic pictures, extraordinary shapes with intense kaleidoscopic play of colours”. Later, he felt his body was possessed by a demon and his neighbour was a witch. This was the first demonstration that a man-made substance can provoke psychosis. Since that time an increasing number of drugs have been found to have the capacity to increase risk of, or directly cause, psychosis both acute and chronic, and drug-induced psychosis has become a major public health problem.

In this chapter, we will review the major types of drug-induced psychosis, as well as the rising availability, and use, of psychotogenic drugs; the latter of course have been followed by the increase in drug-induced psychosis. We will start with the two types of substances which most commonly cause psychosis, cannabis and stimulants. We will also briefly discuss drugs which less commonly cause psychosis including the huge range of new psychoactive substances that have emerged including synthetic cannabinoids, and cathinone derivatives. In this short paper we cannot discuss all of these in detail. Therefore, the interested reader is referred to Murray et al. [1] and to Fiorentini et al. [2].

## Cannabis and the synthetic cannabinoids

There had been reports that cannabis use could induce psychosis for over 150 years but the first convincing evidence came in 1987 from the Swedish Conscripts Study in which 45,570 conscripts into the army were followed-up for 15 years. The risk for schizophrenia was 6 times higher in those who had smoked cannabis more than 50 times at the initial interview at age 18 years of age [3].

Obtaining appropriate data to replicate this study was not easy but 15 years later, other epidemiological studies on cannabis use and schizophrenia started to be published, and now consistent evidence supports a causal association between heavy cannabis use and the risk of psychotic symptoms and illness. Thus, Sideli et al. [4] stated that “… systematic reviews and methodologically robust studies have confirmed the important role of cannabis use in the initiation and persistence of psychotic disorders.” Those individuals who have a family history of psychosis and those who begin using cannabis in adolescence (which is only too common) appear especially vulnerable.

Critics proposed a number of alternative explanations to the causal hypothesis, but one by one, these were rebutted. Taking into account other drug use or prior psychological deviancy does not negate the effect of cannabis. It was hypothesized that patients may use cannabis to counteract symptoms of psychosis, or even allay anxiety in the prodrome, but empirical data do not support this. One suggestion was that those who are genetically predisposed to schizophrenia might be more likely to use cannabis than the rest of the population. Some, but not all, studies suggest that a small proportion of variance in initiating cannabis use may be explained by such genetic predisposition. However, the most recent large study [5] shows that there if there is any such effect, it is very small and cannot explain the strong association between the daily use of high-potency cannabis and later psychosis.

The distinctive clinical picture presented by patients with cannabis-induced psychosis has become clear in recent years. Such patients tend to have higher current and premorbid intelligence quotient (IQ) than other psychotic patients and better premorbid social function. They present earlier and have more positive symptoms especially paranoid delusions. Continued cannabis use after onset of psychosis predicts higher relapse rates, longer hospital admissions, and more severe positive symptoms than for individuals who discontinue cannabis use and those who are nonusers [6].

Delta-9-tetrahydrocannabinol (THC) is the main psychoactive ingredient in cannabis, responsible for the “high” that those who use cannabis experience. When given experimentally, it can produce transient psychotic symptoms [7] and impair working and episodic memory via stimulation of CB_1_ receptors, targets for the endogenous cannabinoid (endocannabinoid [eCB]) transmitters. CB_1_ receptors are widely distributed throughout the brain; they are primarily expressed on glutamate and GABA-ergic terminals, and upon stimulation by cannabinoids, they depress presynaptic glutamate or GABA release. The glutamate and GABA inputs to the dopamine neurons in the ventral midbrain express CB_1_ receptors, and therefore exogenous cannabinoids alter the balance of excitation and inhibition reaching dopamine cells. The most commonly documented effect is an increase in firing with attendant elevations of dopamine release at downstream forebrain territories [1]. Other cannabinoid derivatives have increased in popularity recently. The most common, delta‑8 THC, is typically manufactured from hemp-derived cannabidiol (CBD), and has similar but milder psychoactive effects than the typical delta‑9 THC.

The more potent the cannabis used, the greater the risk of psychotic illness. The proportion of THC in plant cannabis has risen from 3% in many traditional herbal forms to an average of about 15–20% in Europe and North America. In Europe, cannabis is considered high potency if it contains more than 10% THC. Di Forti et al. [8] demonstrated the dose–response relationship: in those who used high-potency cannabis moderately, the risk doubled; with heavy use, it increased 5‑fold; and daily, it increased up to 9‑fold. Di Forti et al. [8] found that the incidence rate of psychosis in 11 areas across 5 countries in Europe was positively correlated with the prevalence of daily cannabis use in the general population in each site; 30% of new cases of psychosis in London and 50% in Amsterdam were secondary to cannabis use, with much smaller proportions in those cities where high potency cannabis was less available. In a similar study, Lee Pow et al. [9] compared the incidence of psychosis in a rural area in India, with that in Ibadan in Nigeria, and the island of Trinidad. The incidence was much higher in Trinidad than the other two sites and this appeared consequent upon the greater frequency of cannabis especially high potency cannabis (25% THC is common in Trinidad).

Synthetic cannabinoids: From 2004, a range of synthetic cannabinoids, often termed Spice or K2 or Kush, started to become available. These are full-blown agonists at the CB_1_ receptor and consequently have stronger effects than traditional plant-based cannabis. Initially they were repurposed from experimental drugs developed in pharmacology labs but more recently they have often been produced by modifying drugs that had been made illegal. As they are not detected by common drug screens they are especially popular amongst those who wish to avoid detection, e.g. those in prison or in the army. As they have rarely been tested in animals some of these drugs have occasionally been found to induce acute cardiovascular or other disease [2].

## Stimulants

### Amphetamines

Amphetamine psychosis was first clearly described in the 1950s. Having been taken up into dopamine varicosities, amphetamine displaces dopamine from the intracellular vesicles; it also acts to reverse the direction of the dopamine transporter (DAT) on the cell surface, so that rather than being recycled, dopamine is now actively pumped out of the varicosity onto recipient neurons. Reversibility of the transporter involves an intermediate called TAAR1 (trace amine-associated receptor 1), which has attracted recent attention as a possible target for new antipsychotic drug discovery.

Psychosis secondary to amphetamine abuse was reported from many countries but never became highly prevalent. However, since the 1990s there has been a rapid spread in use of the more addictive methamphetamine. Reports of methamphetamine psychosis came first from Japan and then from Taiwan where Chen et al. [10] examined 163 patients who presented a picture almost identical to that of paranoid schizophrenia except that visual hallucinations appeared more common than one might have expected. Methamphetamine users with a pre-existing psychosis-prone personality were more likely to have developed frank psychosis as were those with a greater familial risk. In most cases the symptoms disappeared following cessation of the drug, but in a minority they did not remit readily, if ever. Psychosis-prone personality and family history were also associated with a more prolonged psychosis.

Methamphetamine use, and its resultant psychosis spread to Thailand, Australia and South Africa [1]. In the latter two countries up to 25% of all people presenting with psychosis in many centres now do so following the use of methamphetamine. Methamphetamine use then spread to North America with psychotic cases being reported from the West Coast of USA and Canada, before moving eastwards. Tardelli et al. [11] noted a “dramatic increase in psychotic disorders in the context of amphetamines” admitted to hospitals in Toronto between 2014 and 2021. Cases then started being reported from UK and Europe, and the Middle East but methamphetamine psychosis has not yet become common in either.

### Cocaine

Cocaine binds to the dopamine transporter, and also inhibits serotonin and noradrenaline reuptake. Psychotic symptoms particularly paranoia and suspiciousness as well hallucinations including visual hallucinations can occur though possibly less frequently than one might imagine given the frequency of its use, and dependence. There appears to be a dose–response effect, and possibly early use may be more risky [2].

### Khat and the cathinones

Khat is grown in the countries of the East Africa and the South-Western Arabian Peninsula, and chewed for its stimulant effect. In Yemen, for example, up to 60% of the males have been reported to chew Khat leaves. Odenwald et al. [12] screened nearly 5000 persons in Somali and identified those with psychotic symptoms. These cases had started to use Khat early in life and had been using Khat almost a decade before psychotic symptoms emerged, often following a binge of use. Although most cases continue to be reported from Ethiopia and neighbouring countries, migration of people then allowed a much wider use of Khat and resultant psychosis [13] including to Europe.

Synthetic cathinones: The most psychoactive component in Khat is cathinone which produces effects analogous to those of amphetamine. In recent decades, synthetic cathinones, commonly referred to as “bath salts”, have become widely used recreationally. They are often obtained via the internet. One of the most popular synthetic cathinones, is mephedrone, sometimes known as “Meow meow” because because its chemical name is methylmethcathinone. It has been made illegal in a number of countries with a decline in its use. However, given the frequency of the use of synthetic cathinones, resultant psychosis is relatively uncommon, a fact confirmed by Dasmwani et al. [14] who conducted a systematic review of cases.

### Entactogens

3,4-Methylenedioxyamphetamine (MDA) derivatives are amphetamine-like drugs which are now termed “empathogens” or “entactogens”. The most popular, MDMA (ectasy), produces a psychostimulant effect together with euphoria and often a feeling of benevolence towards others. The proportion of users who develop psychosis is very low and indeed the occasional occurrence may be a coincidence.

## Ketamine

Phencyclidine (PCP) is a glutamate antagonist, and when administered to healthy volunteers produces disturbances in body image, affect and thinking similar to psychosis. PCP achieved some popularity in the 1960s and 1970s as a recreational drug sometimes termed “angel dust”.

Then, ketamine, which has similar glutamatergic effects was also introduced as an anaesthetic, and later reports of ketamine-induced psychosis started to appear. Chen et al. [15] reported a series of cases of chronic psychosis associated with long-term ketamine use. The picture included positive symptoms such as hallucinations, delusions and thought disorder, but also withdrawal, decreased motivation and dissociation. Thus, negative symptoms are more common with PCP and ketamine than other psychotogenic drugs.

By obstructing the channel pore of the glutamate N-methyl-D-aspartate (NMDA) receptor, ketamine prevents the otherwise rapid influx of Ca^2+^ into the postsynaptic neuron. NMDA receptors are enriched on the dendritic spines of neurons in areas of the brain such as the cortex and the hippocampus. In normal physiology, the opening of NMDA channels is the primary event leading to the strengthening of a synaptic connection, for a very brief period as in working memory or sometimes as a permanent memory trace. In an acute setting, ketamine can disrupt the normal flow of working memory leading to a marked distortion of reality processing, alogia, withdrawal and sometimes bizarre psychotic experiences.

The evidence that ketamine could cause both positive and negative psychotic symptoms gave rise to the idea that it provided a model of schizophrenia and indeed to the “glutamate model of psychosis”.

## Hallucinogens

In the two decades following Hoffman’s synthesis of lysergic acid diethylamide (LSD), there was interest in the idea that this drug provided a model of schizophrenia. As it produces its psychotogenic effects by stimulating 5HT_2A_ receptors on central neurons, researchers discussed the possibility of a “serotonergic model of psychosis”. However, the hallucinations tend to be visual rather than auditory. Much evidence suggests that use can produce a psychosis which is self-limiting, and goes away with cessation of drug taking. A persistent psychosis is uncommon [16].

Other tryptamines with similar effects include DMT, psilocybin (4-phosphoryloxy-DMT); while the phenethylamines include mescaline and many synthetic hallucinogens.

## The incoming tide

Use of many psychotogenic drugs has become more common in the last 30 years with consequent increase in drug-induced psychosis and schizophrenia. There is often confusion between the diagnosis of drug-induced psychosis and schizophrenia. The former is often used to describe patients who have a relatively brief psychosis. However the longer drug use continues, the more that the clinical picture takes on the characteristics of schizophrenia. The study of Rognli et al. [17] shows that of those who presented with substance-induced psychosis in Norway, 28% receive a diagnosis of schizophrenia within the next 5 years; the proportion reaches nearly half in those with a cannabis-induced psychosis.

The increase in methamphetamine-induced psychosis in many countries has occurred without any liberalisation of laws. The reasons include the facts that methamphetamine is highly addictive and very easy to make in small “kitchen” labs from ephedrine or pseudoephedrine which are widely available. Concerning Khat, some countries have moved to make its use illegal but the number of derivative synthetic cathinones available on the internet have continued to increase; the latter are not commonly blamed for psychosis. Ketamine remains illegal for recreational use worldwide but it has begun to be prescribed as a treatment for depression, and attitudes to its use have softened in many countries, leading to its increasing recreational use; little is known regarding the effects of this on rates of psychosis. In spite of some alarmist reports, enactogens, such as MDMA whose use greatly increased in the first years of 21st century, do not appear to be common causes of schizophrenia-like psychosis; the same can be said for LSD.

Legalisation of cannabis: Cannabis-induced psychosis has increased in many countries where the drug is still illegal, probably due to the increased availability and potency of the drug. Boydell et al. [18] were the first to show this; the incidence of schizophrenia doubled in London, England, between 1965 and 1999 and much of this appeared to be due to the increased use of cannabis. A Danish registry study found that the proportion of schizophrenia attributable to cannabis use disorder (CUD) increased by four times between 1992 and 2016 [19].

However, there is a worldwide trend toward liberalizing the laws concerning cannabis [20]. Many countries have legalised cannabis for so-called “medicinal” use, while others countries have partly decriminalized its use, e.g. the Netherlands and Portugal. Uruguay legalized recreational cannabis in 2013, as did Canada in 2018. In all, 39 US states have legalised cannabis for “medicinal” use and 24 for recreational use; in some states the criteria for receiving “medicinal” cannabis are so lax that in practice this is often a means to use it recreationally. In the US states that have legalized cannabis, the price has fallen and both cannabis use and dependence have increased, as have accidental overdoses and driving while intoxicated. There is concern about the effects of cannabis on the unborn child, particularly since many US marijuana dispensaries advise women to use cannabis during pregnancy to counteract nausea.

Has liberalisation of recreational cannabis use laws increased rates of psychosis? In Portugal where cannabis was decriminalised in 2001 [21], the proportion of schizophrenia attributed to cannabis use increased tenfold by 2015; this may however have been partly due to greater awareness of the psychotogenic effects of cannabis. Since legalisation for medicinal use in Canada in the early 2000s, the proportion of schizophrenia associated with diagnosed cannabis use disorder (CUD) has increased from 1.6% in 2006 to 9.6% in 2022 [22]; many others are likely to have been heavily using cannabis without a formal diagnosis of CUD; the increase in psychotic presentations has accelerated since legalisation for recreational use in 2018. In those US states that have liberal cannabis laws, a higher proportion of psychosis is associated with cannabis use than in states where cannabis is still illegal. In Colorado, the first US state to legalize recreational use (in 2012), potency has spiralled upwards with 70% THC content in shatter (solid cannabis extract) and wax dabs (soft, honeylike cannabis extract), and the rate of hospital visits for psychosis have greatly increased [23].

Legalization of cannabis production and sale has created a major industry in North America with a strong financial interest in promoting cannabis use [20]. As a result, commercialization of cannabis is now rampant, and an astonishing array of cannabis products in the form of ice cream, cakes, and sweets is available. A recent report estimated annual worldwide legal sales in 2025 of cannabis at $ 68 billion. Tobacco and alcohol companies are buying into the industry. Sophisticated lobbying and marketing by the cannabis industry in North America highlight the tax revenue for governments and the supposed health benefits of “medicinal use”.

Is it inevitable that legalization of recreational cannabis will result in more dependence and psychosis? In theory, it should be possible to legalize cannabis in ways that do not increase potency and prevalence of use but, so far, experience in North America is not encouraging. Governments that legalize cannabis use should monitor the effects on mental health, and devote a proportion of their tax receipts from cannabis to dealing with the health consequences. Most importantly public health campaigns are necessary to advise the population of the risks of heavy use of high potency cannabis. In the absence of this, it seems likely that the increasing commercialization of recreational cannabis will be followed by increases in the incidence of new cases of psychosis and in a few years in the prevalence of chronic schizophrenia.

## References

[1] Murray RM, Paparelli A, Morrison PD, Marconi A, Di Forti M. What can we learn about schizophrenia from studying the human model, drug-induced psychosis? Neuropsychiatr Genet. 2013;162B(7):661–70. 10.1002/ajmg.b.32177 24132898

[2] Fiorentini A, Cantù F, Crisanti C, Cereda G, Oldani L. Brambillo P. Substance-Induced Psychoses: An Updated Literature Review. Front Psychiatry. 2021;23(12):694863. 10.3389/fpsyt.2021.694863.eCollection20213. 10.3389/fpsyt.2021.694863 PMC8732862 35002789

[3] Andreasson S, Allebeck P, Engstrom A, Rydberg U. Cannabis and Schizophrenia: A longitudinal study of Swedish conscripts. Lancet. 1987;26:1483–6. 10.1016/s0140-6736(87)92620-1 2892048

[4] Sideli L, Quigley H, La Cascia C MRM. Cannabis use and the risk for psychosis and affective disorders. J Dual Diagn. 2020;16(1):22–42. 31647377 10.1080/15504263.2019.1674991

[5] Austin-Zimmerman I, Spinazzola E, Quattrone D, Wu-choi B, Trotta G, Li Z. The impact of schizophrenia genetic load and heavy cannabis use on the risk of psychotic disorder in the EU-GEI case-control and UK Biobank studies. Psychol Med. 2024;54(15):4160–72. 10.1017/S0033291724002058. 39637925 10.1017/S0033291724002058 PMC11650186

[6] Schoeler T, Monk A, Sami MB, Klamerus E, Foglia E, Brown R, et al. Continued versus discontinued cannabis use in patients with psychosis: A systematic review and meta-analysis. Lancet Psychiatry. 2016;3(3):215–25. 26777297 10.1016/S2215-0366(15)00363-6

[7] Morrison PD, McKeown Z, Lee TD, Holt DW, Powell JF, Kapur S, Murray RM. The acute effects of synthetic intravenous Delta9-tetrahydrocannabinol on psychosis, mood and cognitive functioning. Psychol Med. 2009;39(10):1607–16. https://doi.org/10.1080/15504263.2019.1674991. 19335936 10.1017/S0033291709005522

[8] Di Forti M, Quattrone D, TP F, Jongsma H, Kirkbride J, Morgan C, Murray RM. The contribution of cannabis use to variation in the incidence of psychotic disorder across Europe (EU-GEI): a multicentre case-control study. Lancet Psychiatry. 2019;6(5):427–36. 10.1016/S2215-0366(19)30048-3. 30902669 10.1016/S2215-0366(19)30048-3 PMC7646282

[9] Pow LJ, Donald C, Di Forti M, Roberts Weiss Ayinde THAO. Cannabis use and psychotic disorders in diverse settings in the Global South: findings from INTREPID II. Psychol Med. 2023;53(15):7062–9. 10.1017/S0033291723000399. 36951137 10.1017/S0033291723000399 PMC10719629

[10] Chen CK, Lin SK, Sham PC, Ball D, Loh EW, Hsiao CC, Chiang YL, Ree SC, Lee CH, Murray RM. Pre-morbid characteristics and co-morbidity of methamphetamine users with and without psychosis. Psychol Med. 2003;33:1407–14. 14672249 10.1017/s0033291703008353

[11] Tardelli VS, Johnstone S, Xu B, Kim S, Kim H, Gratzer D, George TP, Le Foll B, Castle DJ. Marked Increase in Amphetamine-Related Emergency Department Visits and Inpatient Admissions in Toronto, Canada, 2014–2021. Can J Psychiatry. 2023;68(4):249–56. 36809914 10.1177/07067437221125302 PMC10037744

[12] Odenwald M, Neuner F, Schauer M, Elbert T, Catani C, Lingenfelder B, Hinkel H, Häfner H, Rockstroh B. Khat use as risk factor for psychotic disorders: a cross-sectional and case-control study in Somalia. BMC Med. 2005;12(3):5. 10.1186/1741-7015-3-5. 10.1186/1741-7015-3-5 PMC554104 15707502

[13] Edwards B, Atkins N. Exploring the association between khat use and psychiatric symptoms: a systematic review. Bmj Open. 2022;12:e61865. 10.1136/bmjopen-2022-061865. 35879018 10.1136/bmjopen-2022-061865 PMC9328084

[14] Daswani RR, Choles C, Kim DD. Barr AM A systematic review and meta-analysis of synthetic cathinone use and psychosis. Psychopharmacology. 2024;241(5):1–22. 38446172 10.1007/s00213-024-06569-x

[15] Cheng W‑J, Chen C‑H, Chen C‑K, Huang M‑C, Pietrzak RH, Krystal JH, Xu K. Similar psychotic and cognitive profile between ketamine dependence with persistent psychosis and schizophrenia. Schis Res. 2018;199:313–8. 10.1016/j.schres.2018.02.049. 10.1016/j.schres.2018.02.049 29510925

[16] Rucker JJH, Iliff J, Nutt DJ. Psychiatry & the psychedelic drugs. Past, present & future. Neuropharmacology. 2018;142:200–18. 10.1016/j.neuropharm.2017.12.040. 29284138 10.1016/j.neuropharm.2017.12.040

[17] Rognli EB, Heiberg IH, Jacobsen BK, et al. Transition from substance-induced psychosis to schizophrenia spectrum disorder or bipolar disorder. Am J Psychiatry. 2023;180:437–44. 37132221 10.1176/appi.ajp.22010076

[18] Boydell J, van Os J, Caspi A, et al. Trends in cannabis use prior to first presentation with schizophrenia, in South-East London between 1965 and 1999. Psychol Med. 2006;36(10):1441–6. 10.1017/S0033291706008440. 16854250 10.1017/S0033291706008440

[19] Hjorthøj C, Posselt CM, Nordentoft M. Development over time of the population-attributable risk fraction for cannabis use disorder in schizophrenia in Denmark. Jama Psychiatry. 2021;78(9):1013–9. 34287621 10.1001/jamapsychiatry.2021.1471 PMC8295899

[20] Murray RM, Hall W. Will Legalization and Commercialization of Cannabis Use Increase the Incidence and Prevalence of Psychosis? Jama Psychiatry. 2020;77(8):777–8. 10.1001/jamapsychiatry.2020.0339. 32267480 10.1001/jamapsychiatry.2020.0339

[21] Gonçalves-Pinho M, Bragança M, Freitas A. Psychotic disorders hospitalizations associated with cannabis abuse or dependence: a nationwide big data analysis. Int J Methods Psychiatr Res. 2019; 10.1002/mpr.1813. Published online December 5. 31808250 10.1002/mpr.1813 PMC7051837

[22] Myran DT, Pugliese M, Harrison LD, Solmi M, Anderson KK, Fiedorowicz JG, et al. Changes in incident schizophrenia diagnoses associated with cannabis use disorder after cannabis legalization. Jama Netw Open. 2025;8(2):e2457868–e2457868. 39903464 10.1001/jamanetworkopen.2024.57868 PMC11795325

[23] Joshi S, Snyder KM, Thurstone C, Rivera BD, Feldman J, Cerdá M, et al. Changes in psychosis-related emergency department and hospitalization rates among youth following cannabis legalization in Colorado. Drug Alcohol Depend. 2025; 112719. 10.1016/j.drugalcdep.2025.112719 PMC12188688 40451017

